# Anti-*Helicobacter pylori *activity and immunostimulatory effect of extracts from *Byrsonima crassa *Nied. (Malpighiaceae)

**DOI:** 10.1186/1472-6882-9-2

**Published:** 2009-01-16

**Authors:** Cibele Bonacorsi, Maria Stella G Raddi, Iracilda Z Carlos, Miriam Sannomiya, Wagner Vilegas

**Affiliations:** 1 Departamento de Análises Clínicas, Faculdade de Ciências Farmacêuticas, Universidade Estadual Paulista (UNESP), Rua Expedicionários do Brasil 1621, CEP14801-960, Araraquara, SP, Brazil; 2 Departamento de Química Orgânica, Instituto de Química, Universidade Estadual Paulista (UNESP), Rua Franciso Degni s/n, CEP 14800-900, Araraquara, SP, Brazil

## Abstract

**Background:**

Several *in vitro *studies have looked at the effect of medicinal plant extracts against *Helicobacter pylori *(*H. pylori*). Regardless of the popular use of *Byrsonima crassa *(*B. crassa*) as antiemetic, diuretic, febrifuge, to treat diarrhea, gastritis and ulcers, there is no data on its effects against *H. pylori*. In this study, we evaluated the anti-*H. pylori *of *B. crassa *leaves extracts and its effects on reactive oxygen/nitrogen intermediates induction by murine peritoneal macrophages.

**Methods:**

The minimal inhibitory concentration (MIC) was determined by broth microdilution method and the production of hydrogen peroxide (H_2_O_2_) and nitric oxide (NO) by the horseradish peroxidase-dependent oxidation of phenol red and Griess reaction, respectively.

**Results:**

The methanolic (MeOH) and chloroformic (CHCl_3_) extracts inhibit, *in vitro*, the growth of *H. pylori *with MIC value of 1024 μg/ml. The MeOH extract induced the production H_2_O_2 _and NO, but CHCl_3 _extract only NO.

**Conclusion:**

Based in our results, *B. crassa *can be considered a source of compounds with anti-*H. pylori *activity, but its use should be done with caution in treatment of the gastritis and peptic ulcers, since the reactive oxygen/nitrogen intermediates are involved in the pathogenesis of gastric mucosal injury induced by ulcerogenic agents and *H. pylori *infections.

## Background

*Helicobacter pylori *(*H. pylori*) is a spiral-shaped bacterium that colonizes the stomach of the half of all people worldwide [1]. Once a person is infected, the organism can live in the stomach indefinitely and may not cause clinical illness. It is still not clear how *H. pylori *are transmitted or why some people infected with its bacteria become sick and others do not [2]. Studies have also shown an association between long-term infection with *H. pylori *and the development of gastric adenocarcinoma [3,4].

Therapy for *H. pylori *infection consists of 1–2 weeks of one or two effective antibiotics, such as amoxicillin, tetracycline, metronidazole, or clarithromycin, plus either ranitidine bismuth citrate, bismuth subsalicylate, or a proton pump inhibitor [5]. Many clinical treatment trials involving patients with *H. pylori *infection and gastric or duodenal ulcers show that curing the infection is associated with a marked reduction in ulcer recurrence rates [6]. However, eradication by the triple therapy is not always successful and the acquisition by *H. pylori *resistant to antibiotics could represent a serious problem that may reduce treatment efficacy [7]. Considering that eradication therapies can be ineffective and undesirable side effects may occur, the search for new drugs for the development of alternative therapies is very important [1]. Plant extracts are among the attractive sources of new drugs and have been shown to produce promising results in the treatment of gastric ulcers [8-11].

The genus *Byrsonima*, which is composed of approximately 150 species, belongs to the Malpighiaceae family and is widely distributed throughout tropical America [12,13]. This family is constituted by approximately 800 species distributed in 60 genera and about 50% of these species are concentrated in Brazil [14]. In traditional Brazilian medicine, *Byrsonima crassa *(*B. crassa*) is used as antiemetic, diuretic, febrifuge, to treat diarrhea, gastritis and ulcer [15].

The potential antiulcerogenic of *B. crassa *leaves extracts were demonstrated by Sannomiya *et al*. [16]. The results of this research showed that methanolic (MeOH) extract provided better gastroprotective activity than chloroformic (CHCl_3_) extract. The presence of amentoflavone, quercetin derivatives and catechins in the MeOH extract were suggested to contribute for the gastroprotective activity since these compounds were reported to inhibit lipid peroxidation as well as possess a very potent antioxidant activity [16,17].

Several *in vitro *studies have looked at the effect of medicinal plant extracts against *H. pylori *[18-21]. This bacteria induces inflammation, infiltration and activation of immune cells, accumulation of reactive oxygen species, and oxidative DNA damage in the gastric mucosa [22-24]. The antimicrobial compounds from plants may inhibit bacterial growth by different mechanisms than those presently used antimicrobials, and could therefore be of clinical value in the treatment of resistant microbial strains, including *H. pylori *[25].

Despite of the popular use of *B. crassa *as a medicinal plant, there is no data on its antimicrobial activity and immunostimulatory effects. In this study, we evaluated, in vitro, the anti-*H. pylori *of *B. crassa *leaves extracts (MeOH and CHCl_3_) and its immunostimulatory effects in murine immune system by determination of oxygen (H_2_O_2_) and nitrogen (NO) intermediates reactive.

## Methods

### Plant material

*B. crassa *Nied. (Malpighiaceae) leaves were collected at Porto Nacional, Tocantins State, Brazil and authenticated by Prof. Eduardo Ribeiro dos Santos. A voucher specimen (Nr. 3377) was deposited at the Herbarium of the Tocantins University.

### Extraction and preparation of extract solutions

The aerial parts (2.0 kg of leaves) obtained were dried (at 40°C for 4 days) and powdered. The dry powdered material was macerated three times with 2 liters of chloroform and methanol successively at room temperature and left for 48 h in the respective solvent. The solvents were filtered and evaporated at 60°C under reduced pressure providing CHCl_3 _(53.8 g) and MeOH (158.3 g) extracts. The yields (w/w) for the CHCl_3 _and MeOH extracts from the air-dried and powdered leaves of *B. crassa *leaves were 2.7 and 7.9%, respectively [16]. Stock solutions of plant extracts (50 mg/ml) were prepared in dimethyl sulfoxide (DMSO) and stored at -20°C. Dilutions of the stock solutions were made in Brain Heart Infusion (BHI) plus 10% fetal bovine serum (FBS) for antimicrobial activity and in potassium phosphate buffer or RPMI-1640 medium for measurement of H_2_O_2 _and NO production by peritoneal macrophages. Fresh solutions were prepared for each experiment.

### Bacterial strain

*H. pylori *ATCC 43504, metronidazole resistant (MtzR) and amoxicillin susceptible (AmxS), was obtained from the American Type Culture Collection (Manassas, VA, USA). The bacterium was cultured in Columbia agar containing 5% sheep blood at 36–37°C for 3 days, in 5% O_2_, 10% CO_2_, 85% N_2 _atmosphere.

### Antimicrobial activity

The wells of a 96-well microplate were filled with 100 μl of various concentrations of the extracts. Same volume of *H. pylori *suspension (about 10^6 ^cfu/ml) was added to each well. The absorbance was determined using an automatic ELISA microplate reader (Spectra & Rainbow Readers, Tecan) adjusted at 620 nm. The microplate was incubated at 36–37°C for 3 days, under microaerophilic atmosphere, agitated and the absorbance was read again in the reader at the same wavelength. The absorbencies were compared to the values obtained before incubation to detect an increase in bacterial growth. The lowest concentration of the test extract resulting in inhibition of bacterial growth, at least, more than 90%, was taken as the minimal inhibitory concentration (MIC). Amoxicillin and metronidazole were used as reference antimicrobial.

### Animals

Experiments involving Swiss mice (6–8 weeks old, 18 to 25 g) were performed in accordance with the regulations of Research Ethics Committee (01/2005), Faculty of Pharmaceutical Sciences, Unesp, São Paulo, Brazil.

### Peritoneal macrophages

Resident and thioglycollate-elicited peritoneal exudate cells were obtained from mice following intraperitoneal injection of 3 ml thioglycollate medium (3.0 g/100 ml) and lavage of the peritoneal cavity with 5 ml of 10 mM phosphate-buffered saline (PBS), pH 7.2, 3–4 days later. The proportion of macrophages in the peritoneal exudate was determined by cell staining with May-Gruenwald-Giemsa. Cell preparations contained more than 95% macrophages. The cells were washed twice with PBS and resuspended in appropriate medium for each test.

### Macrophages viability

For the determination of the concentrations of extracts that do not cause cell death, the cytotoxic assay was performed as described. Macrophages (10^6 ^cells/ml) were suspended in RPMI-1640 containing 5% heat-inactivated FBS, 100 IU/ml penicillin, 100 μg/ml streptomycin and 50 mM 2-mercaptoethanol. The suspension (100 μl) was added to each well of a 96-well microplate and the cells were incubated at 37°C in a humidified atmosphere containing 5% CO_2_. After 1 h, the wells were washed and adhering cells exposed to different concentrations of methanolic or chloroformic extract for 1 and 24 h. The test was accompanied by a viability positive control (RPMI plus cells) and negative control (RPMI plus extract). Finally, neutral red (NR) assay [26] was performed and the absorbance at 540 nm (reference filter 620 nm) determined using an automatic microplate reader.

### Measurement of H_2_O_2_

Production H_2_O_2 _was measured by the horseradish peroxidase (HRP)-dependent oxidation of phenol red [27]. Macrophages (2 × 10^6 ^cells/ml) were suspended in 10 mM potassium phosphate buffer containing 140 mM NaCl, 5.5 mM dextrose, 0.56 mM phenol red and 0.01 mg/ml HRP, pH 7.4. Briefly, 100 μl of this suspension was added to each of a 96-well culture tissue plate and exposed to methanolic and chloroformic extracts (concentrations of extracts that do not cause cell death), for 1 h (time for the H_2_O_2 _assay) at 37°C in a 5% CO2 atmosphere. The reaction was stopped by the addition of 10 μl of 1 N NaOH and the absorbances were read at 620 nm using a microplate reader. The results are reported as nmol/2 × 10^5 ^cells. The experiment was accompanied by a positive control (buffer plus macrophages and 200 nM phorbol myristate acetate, PMA) and negative control (buffer plus macrophages).

### Measurement of NO

NO synthesis was determined by measuring the accumulation of nitrite, a stable metabolite of NO, using the Griess reaction [28]. Macrophages (100 μl) in at 5 × 10^6 ^cells/ml in RPMI containing 5% heat-inactivated FBS, 100 IU/ml penicillin, 100 μg/ml streptomycin and 50 mM 2-mercaptoethanol, were added to each well of a 96-well cell culture plate and exposed to methanolic and chloroformic extracts (concentrations that do not cause cell death), for 24 h (time for the NO assay) at 37°C in a 5% CO2 atmosphere. After incubation, 50 μl aliquots of culture supernatant were mixed with an equal volume of Griess reagent and incubated at room temperature for 10 min. Absorbance at 540 nm was measured using a microplate reader. The results are reported as μmol/5 × 10^5 ^cells. Each experiment was accompanied by a positive control (RPMI plus macrophages and 10 μg/ml lipopolysaccharide, LPS) and a negative control (RPMI and macrophages).

### Statistical Analysis

The results are expressed as means ± SD (Standard Deviation). All tests were perform in triplicate and repeated at least three times. Statistical difference between groups was determined by one-way analysis of variance (ANOVA). A *p*-value < 0.05 was considered statistically significant.

## Results

**Figure 1:**
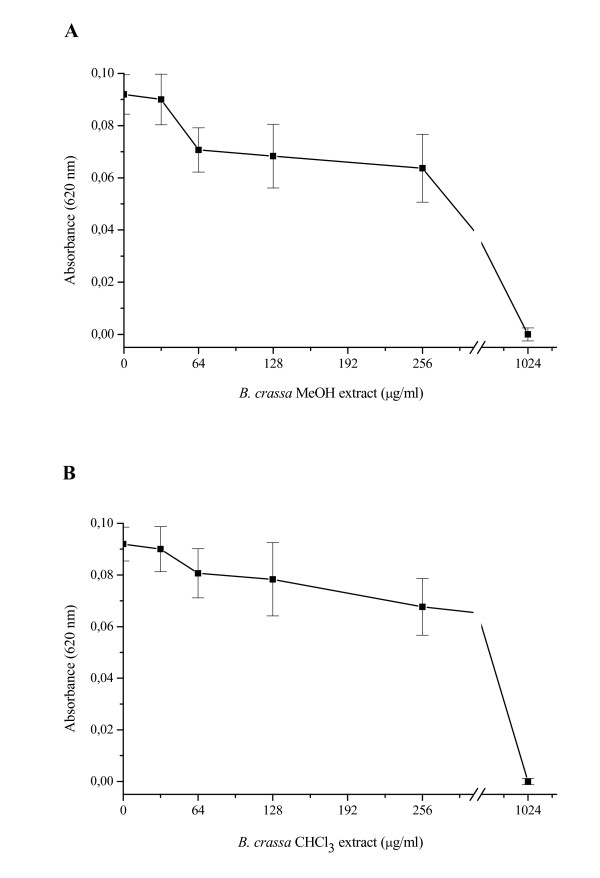
**Effect of the MeOH (A) and CHCl_3 _(B) extracts obtained of *Byrsonima crassa *on growth of *Helicobacter pylori*, following incubation for 72 h**.

**Figure 2:**
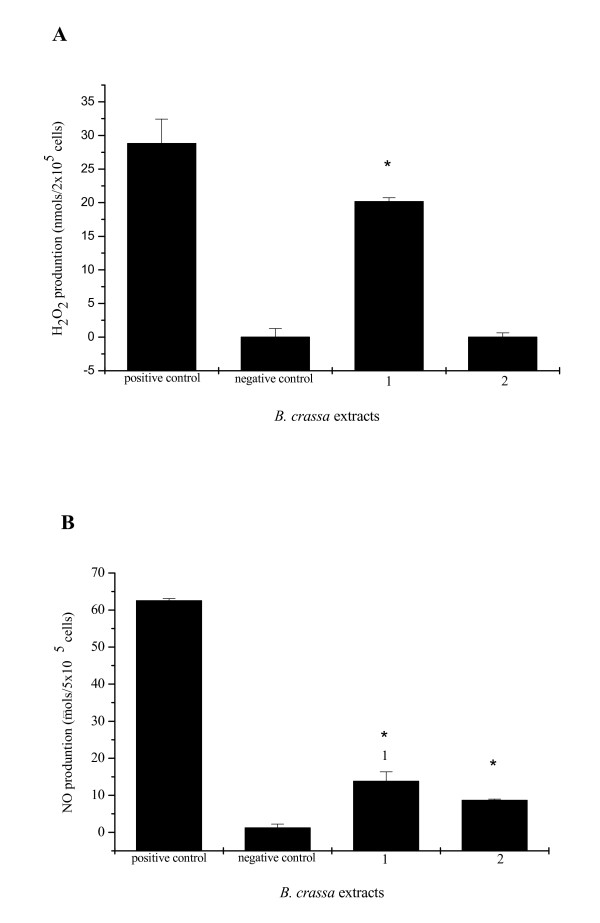
**H_2_O_2 _(A) and NO (B) production from peritoneal macrophages stimulated with 280 μg/ml MeOH (1) and 200 μg/ml CHCl_3 _(2) extracts**. Macrophages were exposed to *Byrsonima crassa *extracts for 1 h (A) and 24 h (B). The results are the mean ± SD of at least three independents experiments carried out in triplicate. *P < 0.05, significantly different from control without stimulating (negative control).

The antibacterial activities of the extracts (MeOH and CHCl_3_) from *B. crassa *against *H. pylori*, using spectrophotometer microdilution assay, are showed in Figure 1. The results demonstrated that both MeOH and CHCl_3 _extract exhibited anti-*H. pylory *activity with MIC value of 1024 μg/ml. The data presented in Figure 2 summarize the release of H_2_O_2 _and NO by murine peritoneal macrophages. The MeOH extract at concentrations of 280 μg/ml (maximum concentration that did not cause cell death) was able to induce the release of 20.16 ± 0.58 nmol/2 × 10^5 ^cells and 13.79 ± 2.58 μmol/5 × 10^5 ^cells H_2_O_2 _and NO, respectively. The CHCl_3 _extract at concentration of 200 μg/ml (maximum concentration that did not cause cell death) just induced the production of NO (8.63 ± 0.35 μmols/5 × 10^5 ^cells).

## Discussion

Many anti-*H. pylori *compounds exhibiting a significant inhibitory effect have been identified from plant materials, among them flavonoids, tannins, terpenes, aromatic aldehydes, alcohols, tannins and catechins [18,29-31]. Based on the popular use of *B. crassa *to treat gastritis and ulcer, we investigated, *in vitro*, the susceptibility of *H. pylori *ATCC 43504 (AmxS and MtzR strain) to MeOH and CHCl_3 _extracts obtained from the leaves of this medicinal plant. Both extracts presented MIC value of 1024 μg/ml. In summary, our results suggest that *B. crassa *produces secondary metabolites with anti-*H. pylori *activity. Probably, the antimicrobial activity demonstrated by extracts may be due to the presence of polyphenolic compounds, such as flavonoids, tannins, and terpenoids described in the phytochemical profile of *B. crassa *[16,32,33], not discarding the possibility of a synergistic effects between substances.

*H. pylori *infection has been associated with generation of oxygen (ROS) and nitrogen (RNS) reactive species, which leads to oxidative stress in gastric mucosa [22,34,35]. This bacterium induces infiltration and activation of phagocytes, which produce inflammatory mediators, cytokines, ROS and RNS. To avoid the negative effects of ROS, *H. pylori*, like many other bacteria, produces enzymes involved in ROS scavenging, such as catalase and superoxide dismutase. *H. pylori *also activates inducible nitric oxide synthase in the gastric mucosa, which is associated with epithelial cell injury and apoptosis [2]. No evidence was found for a role of free radicals in the pathogenesis of gastric mucosal injury in cases unrelated to *H. pylori *infection [35].

Macrophages are widely distributed in different tissues and play an essential role in the development of the specific and nonspecific immune response. These cells can be activated by a variety of stimuli as bacterial components, cytokines and chemicals. Once activated, macrophages produce and release numerous secretors products including several cytokines, inorganic reactive radicals, reactive oxygen intermediates (ROI) and reactive nitrogen intermediates (RNI) with biological activities [36]. Hydrogen peroxide (H_2_O_2_) and nitric oxide (NO) are important in cell signaling and they are effectors molecules for microbicidal and cytotoxic response of macrophages after stimulation [37]. If ROI and RNI may be considered as beneficial intermediates (with respect to its microbicidal and tumoricidal activities), it also can become destructive for the host tissue in certain conditions [38]. NO and reactive oxygen species affect virtually every step of the development of inflammation.

The immunomodulatory activity of several plants compounds has already been described [39]. *Davilla elliptica *chloroform extract triggered the production of H_2_O_2_, NO and tumor necrosis factor-alpha in a dose-dependent manner into cultured macrophages [40] whereas inhibitory effects in H2O2 and NO production by ethyl acetate fraction from *Alchornea glandulosa *were demonstrated [41]. In particular, those plants that reduce the formation of NO may be beneficial in pathophysiological conditions where excessive production of NO is a contributory factor.

In this study, we demonstrated that extracts of *B. crassa *induced the production H_2_O_2 _and NO. Agents that reduce the formation of NO may be beneficial in *H. pylori *infections since an excessive production of NO is an agravating factor in this condition. Finally, increased production of free radicals has been demonstrated to occur during the gastrointestinal metabolism of xenobiotics, which may lead to intestinal disorders [42].

Despite of the antiulcerogenic effect exhibited by *B. crassa *associated with HCl/ethanol induced gastric ulcers [16] and inhibitory activity against *H. pylori*, an immunostimulatory effect on the liberation of H_2_O_2 _and NO by *B. crassa *leaves was demonstrated. Based in these results, *B. crassa *can be considered a source of compounds with anti-*H. pylori *activity, but its use should be done with caution in treatment of the gastritis and peptic ulcers, since the reactive oxygen/nitrogen intermediates are involved in the pathogenesis of gastric mucosal injury induced ulcerogenic agents and *H. pylori *infections.

## Competing interests

The authors declare that they have no competing interests.

## Authors' contributions

MS and WV have been involved in the obtaining the extracts. IZC performed the immunological assays. CB and MSGR carried out the cell viability, antimicrobial experiments and preparation of the manuscript. All authors read and approved the final manuscript.

## Pre-publication history

The pre-publication history for this paper can be accessed here:


